# Surgical Castration in Hormone-Refractory Metastatic Prostate Cancer Patients Can Be an Alternative for Medical Castration

**DOI:** 10.1155/2012/979154

**Published:** 2011-06-15

**Authors:** Masayoshi Zaitsu, Mariko Yamanoi, Koji Mikami, Yuta Takeshima, Naohiko Okamoto, Sadao Imao, Akiko Tonooka, Takumi Takeuchi

**Affiliations:** ^1^Department of Urology, Kanto Rosai Hospital, 1-1 Kizukisumiyoshi-cho, Nakahara-ku, Kawasaki 211-8510, Japan; ^2^Department of Pathology, Kanto Rosai Hospital, 1-1 Kizukisumiyoshi-cho, Nakahara-ku, Kawasaki 211-8510, Japan

## Abstract

*Background*. Most patients with metastatic prostate cancer are endocrinologically treated with LHRH agonist, but finally castration-refractory and hormone-refractory cancers occur. Serum testosterone levels get low to “the castration level” by LHRH agonists but may not get low enough against castration-refractory prostate cancer. *Methods*. As case series, twelve patients suffering from hormone-refractory prostate cancer continuously on LHRH agonist underwent surgical castration. Additionally, one hundred and thirty-nine prostate cancer patients on LHRH agonist or surgical castration were tested for serum total testosterone levels. *Results*. Surgical castration caused decrease in serum PSA in one out of 12 hormone-refractory prostate cancer patients with PSA reduction rate 74%. Serum total testosterone levels were below the sensitivity threshold (0.05 ng/mL) in 40 of 89 (44.9%) medically castrated patients and 33 of 50 (66.0%) surgically castrated patients (*P* = .20). *Conclusion*. Even hormone-refractory prostate cancer patients are candidates for surgical castration because of endocrinological, oncological, and economical reasons.

## 1. Background

Prostate cancer is the most prevalent cancer and the second ranked malignancy which causes death among males in the United States. In Japan as well, the prevalence and death rate of prostate cancer is recently increasing (http://ganjoho.ncc.go.jp/pro/statistics/gdb_trend.html?1%1). Organ confined and minimally invasive prostate cancer can be treated with local therapy such as radical prostatectomy, external beam radiation, and brachytherapy with excellent prognosis. Cryotherapy and high-intensity focused ultrasound for the treatment of localized prostate cancer are also under vigorous investigation with promising initial results. On the contrary, metastatic prostate cancer is usually treated primarily with androgen deprivation therapy with or without antiandrogens, leading to good to fair cancer control, but finally resulting in hormone-refractory cancer, cancer relapse, and cancer death. By definition, castration-refractory prostate cancer responds to secondary hormonal manipulations, including antiandrogen withdrawal, estrogens, and corticosteroids, while true hormone-refractory prostate cancer is resistant to all hormonal measures (Guidelines on prostate cancer, European Association of Urology 2010: http://www.uroweb.org/gls/pdf/Prostate%20Cancer%202010%20June%2017th.pdf).

Androgen deprivation therapy to benign prostatic hypertrophy was first reported by White in 1895 [1]. In 1941, Huggins and Hodges successfully applied androgen deprivation therapy to metastatic prostate cancer patients in the form of surgical castration [2]. In the early 1980s, LHRH agonists were introduced [3] in the endocrinological treatment of advanced prostate cancer, and the oncological outcomes of surgical castration by bilateral orchidectomy and medical castration by LHRH agonists are now generally regarded as the similar. Nevertheless, there are reports indicating that LHRH agonists fail to achieve castrate levels of testosterone [4–6]. According to a nonsystematic review of the literature, castration level of less than 0.5 ng/mL was yielded in 95–98.8% by leuprolide, and that less than 0.2 ng/mL was in 87–92% by leuprolide and in 96% by goserelin [7]. Generally accepted definition of the cut-off point of castration level 0.5 ng/mL was determined by the lower detection limit of assay methods developed in the late 1960s and early 1970s [8] but not used anymore. Despite advances in methodology with more accurate lower limits of detection, the definition of testosterone levels after bilateral orchiectomy, that is, castration level, has not been updated so far. Moreover, there have been no controlled trials which show that the cancer-specific survival of the patients achieving testosterone level less than 0.2 ng/mL or less than 0.05 ng/mL were more favorable compared with those having testosterone level between 0.2 and 0.5 ng/mL.

Olapade-Olaopa et al. presented two cases of prostate cancer who were compliant but resistant to LHRH agonist therapy with normal testosterone levels, while they responded to following surgical castration resulting in low testosterone levels and clinical improvement as well as decrease in PSA [9]. Those cases showed cluster of Leydig cells in the excised atrophic testes. One reason why prostate cancer is resistant to LHRH agonists may be because they occasionally fail to castrate patients sufficiently by unknown reasons in the hypothalamo-pituitary-gonadal axis. Another possibility is that testosterone levels get low to “the castration level” by LHRH agonists but may not get low enough against castration-refractory prostate cancer, as an upregulation of androgen receptor in prostate cancer, and changes in the signal transduction pathways downstream of the receptor get to utilize even little amount of testosterone. Thus, surgical castration can completely eliminate remnant testosterone produced by the testes then may have a possibility in selected cases to control PSA and clinical symptoms of prostate cancer which has been already treated with LHRH agonists. 

Here we show the changes in serum testosterone levels and PSA of hormone-refractory prostate cancer patients who had been treated with LHRH agonists and were surgically castrated. Additionally, we have assessed serum testosterone levels of prostate cancer patients continuously on androgen deprivation treatments, LHRH agonist, and surgical castration, irrespective of their oncological status.

## 2. Methods

Twelve patients suffering from hormone-refractory prostate cancer with multiple bone metastasis underwent surgical castration under spinal or total anesthesia without leaving testicular capsule between October, 2008 and August, 2010. There were no complications associated with surgery. They all were continuously on LHRH agonist, either leuprolide or goserelin, until the time of surgery. All patients had been given at least one antiandrogen (bicalutamide for all, flutamide for 8, chlormadinone acetate for 1), 9 given estramustine, and 10 given corticosteroid during some periods. Seven cases had been previously treated with docetaxel before surgical castration. Their clinical characteristics are listed in Table 1. Serum PSA in all patients and total testosterone in 8 patients were evaluated in pairs before and more than one month after surgical castration. Excised testes in every patient were histologically evaluated especially for the existence of remnant Leydig cells using Hematoxylin-Eosin staining by a board-qualifying pathologist.

One hundred and thirty-nine prostate cancer patients on LHRH agonist (*n* = 89) or surgical castration (*n* = 50) at least for three months and patients who had never experienced (*n* = 31) or who had quitted androgen deprivation therapy (*n* = 10) were tested for their serum total testosterone levels between August and October 2010, regardless of their oncological status. In this study, serum total testosterone was measured by the ECLIA assay (Electrochemiluminescence Immunoassay), and the sensitivity threshold of total testosterone measurement was 0.05 ng/mL.

## 3. Results

PSA declined following surgical castration in 8 cases of hormone-refractory prostate cancer patients on LHRH agonist (Table 1), but only in one case PSA reduction was supposed to be due to surgical castration itself (Figures 2 and 3) on the condition that cases where there exist probable influences of other treatments as docetaxel were excluded. As shown in Table 1, surgical castration made detectable serum testosterone in 4 cases undetectable, while in one case detectable serum testosterone remained detectable (even increased). In 3 cases, serum testosterone had been already undetectable with LHRH agonist. The postorchiectomy rate of cases with serum total testosterone less than 0.05 ng/mL was 87.5% compared with 37.5% before orchiectomy (*P* = .039 by the chi-square test). In the case which showed 74% PSA reduction due to surgical castration, presurgical serum testosterone was 0.07 ng/mL, but post-surgical one was unfortunately unavailable.

Leydig cells were not identified in excised testes of hormone-refractory prostate cancer patients by the ordinary examination of histological specimens.

**Figure 1:**
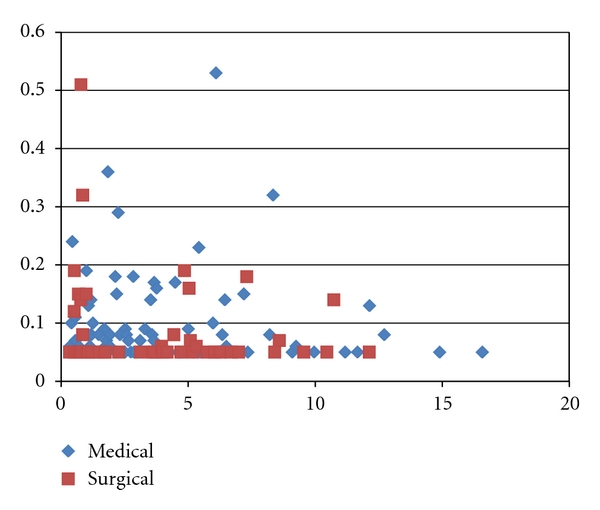
Serum testosterone levels in prostate cancer patients continuously on LHRH agonist or surgical castration. Medical: LHRH agonist, Surgical: surgical castration, vertical line: serum testosterone level (ng/mL), horizontal line: time after induction of LHRH agonist/surgical castration (years).

**Figure 2:**
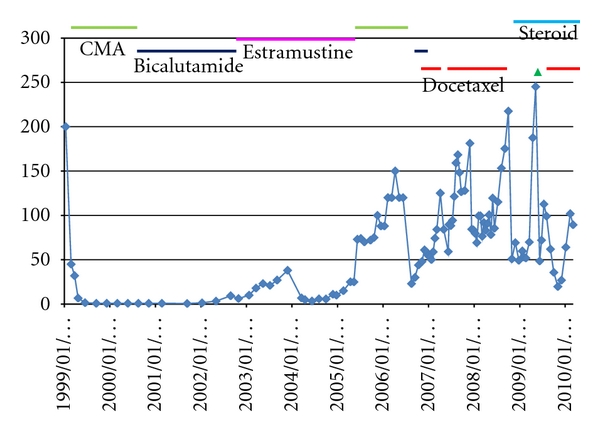
PSA course of a hormone-refractory prostate cancer patient whose PSA declined due to surgical castration. *▵*: surgical castration, CMA: chlormadinone acetate, vertical line: PSA (ng/mL), horizontal line: date. Before surgical castration, dose of docetaxel every other week was 35 mg/m^2^ per course.

In patients on LHRH agonist or surgical castration at least for three months, the ordinary castration levels of serum testosterone (<0.5 ng/mL and <0.2 ng/mL) were achieved in 98.9% and 93.3%, respectively, by medical castration and in 98.0% and 96.0% by surgical castration with the median follow-up period 2.9 years. Serum total testosterone levels were below the sensitivity threshold (0.05 ng/mL) in 40 of 89 (44.9%) medically castrated patients and 33 of 50 (66.0%) surgically castrated patients as shown in Figure 1 (not statistically significant by the chi-square test; *P* = .20). Median serum testosterone levels in patients (age: 56–78 yrs) without experience of androgen deprivation therapy was 4.02 ng/mL (1.61–25.24). Serum testosterone levels in patients (age: 65–77 yrs) who had quitted androgen deprivation therapy was 0.07–5.50 ng/mL (median: 1.49) with time after cessation of androgen deprivation therapy 0.2–5.1 years.

## 4. Discussion

Levels of serum total testosterone in prostate cancer patients on medical and surgical castration were grossly comparable in this study. Surprisingly, serum testosterone above the sensitivity threshold was observed even in patients who had undergone surgical bilateral orchiectomy more than five years before. The source of testosterone is basically unknown in such cases. Extratesticular synthesis of androstenedione and testosterone was reported to be below 0.2%, but detected in liver, kidney, and the gastroduodenal tract in rats [10]. There is possibility that testosterone production is upregulated in those organs other than testis in patients who were surgically castrated and show serum testosterone measurement above the sensitivity threshold. In the clinical setting, the androgen synthesis in the adrenal gland consisting of 10% of circulating androgen is more important for the growth of prostate cancer than extratesticular, extra-adrenal androgen synthesis. Thus, drugs inhibiting adrenal function such as ketoconazole and corticosteroids are in the clinical use.

**Figure 3:**
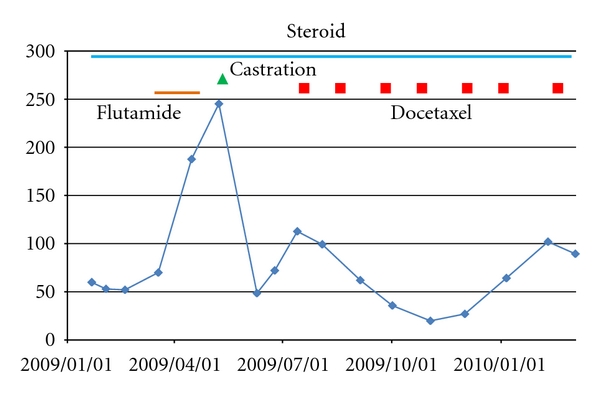
PSA course after 2009/1/1 of the patient described in Figure 2 is enlarged. After surgical castration, docetaxel (70 mg/m^2^ per course every three weeks) was administered together with zoledronic acid (4 mg/body).

Surgical castration in patients who have been on medical castration may possibly be more effective in controlling prostate cancer when castration level caused surgically can be occasionally more profound than that caused medically. Actually in this study, one of twelve hormone-refractory prostate cancer patients with multiple bone metastasis, who were on LHRH agonist, showed 74% reduction of PSA following surgical castration. There may be a question if this case was really hormone refractory or otherwise castration refractory, because hormone-refractory prostate cancer is not controlled anymore by hormonal manipulations by definition. However, it seems reasonable to classify the case into hormone refractory as it failed to control cancer with antiandrogens, estramustine, and corticosteroid. Complete eradication of testosterone by surgical castration may occasionally be effective even to “hormone-refractory” prostate cancer. There is a little possibility that flutamide, which was administered for forty days just before surgical castration without any effects, accelerated PSA increase, and cessation of it returned PSA to the preadministration level as shown in Figure 3. But considering that there was no preceding administration of flutamide and shortness of the duration of flutamide prescription, it was not very probable that there existed mutations in androgen receptor which would drive cancer growth as flutamide binds to the receptor as an agonistic ligand.

In reality, changes in serum testosterone levels of those patients undergoing surgical castration were inconstant, as some showed decrease with surgical castration while others not. In some of the latter cases, serum testosterone levels were already low enough (<0.05 ng/mL) with LHRH agonist, then additional decrease in testosterone might have not been demonstrable due to the sensitivity of measurement. The lower threshold of total testosterone measurement is as low as 0.02 ng/mL owing to the recent development of more sensitive assays [11]. If more sensitive total testosterone assays than that with the lower threshold 0.05 ng/mL are used, we may detect changes in serum total testosterone following surgical castration more precisely. Otherwise extratesticular testosterone production might have occurred as stated before in a case with considerable remaining serum testosterone level following surgery.

Standard androgen deprivation by medical castration does not consistently suppress tumoral androgen activity and androgen-dependent gene expression in the prostate microenvironment, leading to adaptive cellular changes allowing prostate cancer cell survival in a low-androgen environment [12]. This may support a medical rationale for indicating surgical castration for prostate cancer patients resistant to LHRH agonist. Interestingly, there is recently a paradoxical observation that the growth of some “androgen sensitive” human prostate cancer cells expressing androgen receptor can be inhibited by supraphysiologic levels of androgens [13]. Considering that, there may be cases where the administration of androgen can paradoxically suppress prostate cancer which has been treated with androgen deprivation therapy and finally converted to “hormone-refractory” cancer.

Administrating LHRH agonists every one or three month is costly compared with surgical castration which is not expensive and requires a hospital stay shorter than a week. In Japan, surgical fee for bilateral orchiectomy for the endocrine treatment of prostate cancer is just 27,700 yen, while 1-month depots of goserelin and leuprolide cost 43,856 and 46,776 yen, respectively, and 3-month depots of those cost 76,883 and 87,548 yen, respectively. In the time of economical recession as Japan is suffering from, it may be reasonable to think of giving surgical castration instead of medical castration to patients with castration-sensitive and castration-refractory prostate cancer who would hardly quit androgen deprivation therapy in the course of their illness. Japan is supposed to be surpassed by China in the Gross Domestic Product (GDP) by the end of 2010.

## 5. Conclusion

In conclusion, even hormone-refractory prostate cancer patients can be candidates for surgical castration because of endocrinological, oncological, and economical reasons.

##  Conflict of Interests 

There are no competing interests.

##  Author's Contributions 

M. Zaitsu conceived of the study, participated in its design and coordination, and carried out acquisition of data. M. Yamanoi participated in acquisition of data. K. Mikami participated in acquisition of data. Y. Takeshima participated in acquisition of data. N. Okamoto participated in acquisition of data. S. Imao participated in acquisition of data. A. Tonooka performed the pathological diagnosis of excised testes. T. Takeuchi participated in the design and coordination of the study, carried out analysis and interpretation of data, and drafted the manuscript. All authors read and approved the final manuscript.

## Figures and Tables

**Table 1:** 

Case No.	Age at orchiectomy	Prebiopsy PSA	Biopsy GS	LHRH agonist administered	Duration of LHRH agonist	PSA		Testosterone		Overall survival
						pre-	post-	pre-	post-	
1	75	69	8	goserelin	3.9	4.9	2.9	0.10	0.05>	14.9
2	80	488	9	leuprolide	2.5	3.3	2.1	0.12	0.05>	10.6
3	90	10.7	9	goserelin	13.8	29.6	14.9	0.14	0.05>	17.5<
4	76	4.7	8	goserelin	7.0	9.6	16.6	0.11	0.05>	13.7<
5	67	315	8	leuprolide	1.6	924	553	0.07	0.51	9.6
6	77	1463	9	leuprolide	2.1	696	914	0.05>	0.05>	10.1
7	78	2.8	7	leuprolide	1.0	12	4.2	0.05>	0.05>	9.7<
8	78	107	8	goserelin	1.7	0.26	0.12	0.05>	0.05>	3.3<
9*	77	200	7	goserelin	10.3	187	48	0.07	NA	10.1
10	80	426	9	goserelin	0.9	34.1	53.7	NA	0.22	14.8
11	69	334	8	leuprolide	3.2	7.0	0.8	NA	NA	26.1<
12	45	191	6	leuprolide	1.3	138	146	NA	NA	4.4

Units of PSA: ng/mL, GS: Gleason score, duration of LHRH agonist: years, units of serum testosterone: ng/mL, overall survival: survival after surgical castration (months), pre-: before surgical castration, post-: after surgical castration,

*: a case where decrease in PSA was supposed to be due to surgical castration.
